# The definition of immigrant status matters: impact of nationality, country of origin, and length of stay in host country on mortality estimates

**DOI:** 10.1186/s12889-019-6555-1

**Published:** 2019-02-28

**Authors:** Luis Andrés Gimeno-Feliu, Amaia Calderón-Larrañaga, Esperanza Díaz, Clara Laguna-Berna, Beatriz Poblador-Plou, Carlos Coscollar-Santaliestra, Alexandra Prados-Torres

**Affiliations:** 1 EpiChron Research Group on Chronic Diseases, Aragón Health Sciences Institute (IACS), IIS Aragón, Miguel Servet University Hospital, Zaragoza, Spain; 2 Aragón Healthcare Service, San Pablo Health Centre, Zaragoza, Spain; 3 Health Services Research on Chronic Patients Network (REDISSEC), Carlos III Health Institute, Madrid, Spain; 4 Department of Medicine, Psychiatry and Dermatology, University of Zaragoza, Zaragoza, Spain; 5 Department of Neurobiology, Care Sciences and Society, Aging Research Center, Karolinska Institutet, Stockholm, Sweden; 6 Department of Global Public Health and Primary Care, Research Group for General Practice, University of Bergen, Bergen, Norway; 7 Norwegian Centre for Minority Health Research, Oslo, Norway

**Keywords:** Emigration and immigration, Mortality, Population groups, Health inequalities, International health, Spain

## Abstract

**Background:**

Mortality is a robust indicator of health and offers valuable insight into the health of immigrants. However, mortality estimates can vary significantly depending on the manner in which immigrant status is defined. Here, we assess the impact of nationality, country of origin, and length of stay in the host country on mortality estimates in an immigrant population in Aragón, Spain.

**Methods:**

Cross-sectional retrospective study of all adult subjects from the EpiChron Cohort in 2011 (*n* = 1,102,544), of whom 146,100 were foreign-born (i.e., according to place of birth) and 127,213 were non-nationals (i.e., according to nationality). Directly standardized death proportions between years 2012–2015 were calculated, taking into account the age distribution of the European population in 2013. Binary logistic regression was used to compare the four-year probability of death.

**Results:**

The age- and sex-standardized number of deaths per 1000 subjects were 45.1 (95%CI 44.7–45.2) for the Spanish-born population, 29.3 (95%CI 26.7–32.1) for the foreign-born population, and 18.4 (95%CI 15.6–21.6) for non-Spanish nationals. Compared with the Spanish-born population, the age- and sex-adjusted likelihood of dying was equally reduced in the foreign-born and non-national populations (OR 0.6; 95%CI 0.5–0.7) when the length of stay was less than 10 years. No significant differences in mortality estimates were detected when the length of stay was over 10 years.

**Conclusions:**

Mortality estimates in immigrant populations were lower than those of the native Spanish population, regardless of the criteria applied. However, the proportion of deaths was lower when immigrant status was defined using nationality instead of country of birth. Age- and sex-standardized death proportions tended to increase with increased length of stay in the host country.

## Background

Migration is a global phenomenon with important implications for health systems [1]. According to the 2018 United Nations Migration Report, the number of migrants worldwide reached 244 million in 2015 and it is expected to continue to grow [2]. In fact, recent years have seen growing interest in the health status and health determinants of immigrant populations [3–6], the impact of their care on health systems [7, 8], and the design of strategies to improve their health [9].

The International Organization for Migration (IOM) defines an international immigrant as a non-national person who is moving into a country for the purpose of settlement [10]. Other authors use more general definitions, e.g., a person who has established a (semi-) permanent new residence in a “place” other than that in which they habitually lived [11]. In this definition, “place” is generally understood as a locality, district, or higher administrative area. Often, the terms immigrant and foreigner or foreign-born are used interchangeably.

However, the different ways of classifying immigrant status can lead to disparate outcomes in studies conducted on these populations. It is therefore important to take into account the definition used when interpreting and comparing the results of different studies. Two main definitions are found in the literature:

- Foreign-born [11]: a person born in a country other than their current country of residence. This defines an invariable status but excludes second and third generations of immigrants since it is independent of the migratory status of their parents/grandparents. This is the most commonly used definition in the literature.
- Foreigner or non-national [10]: a person belonging to, or owing an allegiance to, another state. This status can vary over time and as a function of the legal requirements of each state, which often depend on historical links between countries.

In 1999, Loue et al. [12] argued for the need to clearly define these terms in research studies*.* Regardless of the concept used, we have not found any previous studies that compare the impact on health outcomes of the use of different definitions.

Spain has one of the largest immigrant populations in absolute numbers in Europe. At the time this study was conducted (i.e., 2011), around 6 million immigrants, defined as those born in another country, were living in Spain, accounting for 13% of the total population, and the National Health Service offered universal coverage regardless of immigrants’ legal status [13]. In Aragón, a region in north-eastern Spain with 1.3 million inhabitants, immigrants represented approximately 13% of the total population in 2010, a proportion equal to that of the entire country.

As opposed to the principle of place of birth, Spanish nationality is based on the principle of jus sanguinis. Therefore, people who are born in another country but have Spanish parents or mixed-nationality parents with at least one of them born in Spain would also have a Spanish nationality.

This study aims to explore the impact of different definitions of immigrant status on health outcome estimations. We used data from Aragón collected as part of the Epichron Cohort [14], taking mortality as the health indicator. In this cohort, both place of birth and nationality are registered. In addition, we also assessed the influence of length of stay in the host country, since the health of immigrants tends to worsen with increased time spent in the host country [5, 15–17].

## Methods

The EpiChron Cohort [14] gathers individual-level clinical and administrative data from primary, secondary and tertiary care health records and the health insurance database for almost all residents of Aragón. In this study, the following variables were included for individuals 14 years or older in 2011: age, sex, nationality, country of birth, and duration of residence in Aragon. National-level mortality data for years 2012–2015 was linked at the individual level.

Four-year death proportions were directly standardized taking into account the age distribution of the European population in 2013. Binary logistic regression was used to compare the four-year probability of death among individuals 1) of a foreign nationality (i.e., non-nationals), and/or 2) born outside of Spain (i.e., foreign-born), with respect to Spanish nationals born in Spain, after adjusting for age (as a continuous variable) and sex. Analyses were stratified by length of stay, applying previously used cut-offs: < 5 years, 5–10 years, > 10 years [15, 18]. The term “immigrant” was used generically to describe individuals with a foreign nationality and/or born outside of Spain. These two groups were further sub-classified according to geographic location (Africa, Asia, Eastern Europe, Latin America, and Western Europe & North America).

Statistical analyses were performed using STATA (version 12; StataCorp, College Station, TX, USA). The study was approved by the Ethics Committee for Clinical Investigation of Aragón.

## Results

The population studied consisted of 1,102,544 people 14 years or older. The main sociodemographic data and number of deaths over the four years of follow-up, stratified according to nationality and place of birth, are shown in Table 1. A total of 56,918 deaths were recorded, corresponding to 5.16% of the population studied. Of these, 428 deaths corresponded to non-nationals who were born outside Spain, while 376 corresponded to Spanish nationals who were born outside Spain.

**Table 1:** Sociodemographic data (2011) and number of deaths (2012–2015) in the study population

	Spanish nationals born in Spain	Spanish nationals born abroad	Non-Spanish nationals born abroad	Total
n (%)	956,444 (86.75)	18,387 (1.67)	127,713 (11.58)	1,102,544
Sex (women), n (%)	491,117 (51.35)	9849 (53.57)	58,526 (45.83)	559,492 (50.75)
Age (years), n (%)				
15–44	395,767 (41.38)	9888 (53.78)	100,160 (78.43)	505,815 (45.88)
45–64	298,854 (31.25)	6775 (36.85)	25,327 (19.83)	330,956 (30.02)
65+	261,823 (27.37)	1724 (9.38)	2226 (1.74)	265,773 (24.11)
Age (years), mean (SD)	50.9 (19.9)	43.7 (15.4)	36.4 (11.5)	49.1 (19.6)
Nationality, n (%)				
Spain	956,444 (100.0)	18,387 (100.0)	–	974,831 (88.42)
Africa	–	–	30,497 (23.88)	30,497 (2.77)
Asia	–	–	4668 (3.66)	4668 (0.42)
Eastern Europe	–	–	48,371 (37.87)	48,371 (4.39)
Latin America	–	–	36,517 (28.59)	36,517 (3.31)
Western Europe & North America	–	–	7660 (6.00)	7660 (0.69)
Place of birth, n (%)				
Spain	956,444 (100.0)	–	–	956,444 (86.75)
Africa	–	2647 (14.40)	30,579 (23.94)	33,226 (3.01)
Asia	–	508 (2.76)	4684 (3.67)	5192 (0.47)
Eastern Europe	–	257 (1.40)	48,360 (37.87)	48,617 (4.41)
Latin America	–	10,059 (54.71)	36,883 (28.88)	46,942 (4.26)
Western Europe & North America	–	4916 (26.74)	7207 (5.64)	12,123 (1.10)
Length of stay in Spain, n (%)				
< 5 years	–	1922 (10,45)	77,323 (60,54)	–
≥ 5 years	–	16,465 (89,55)	50,390 (39,46)	–
Length of stay in Spain, mean (SD)	–	9,7 (3,0)	5,0 (2,8)	–
Deaths, n (%)	56,114 (5.87)	376 (2.04)	428 (0.34)	56,918 (5.16)
Deaths in women, n (%)	27,614 (49.21)	183 (48.67)	159 (37.15)	27,956 (49.12)
Deaths by age group, n (%)				
15–44	1027 (1.83)	24 (6.38)	147 (34.35)	1198 (2.10)
45–64	5855 (10.43)	94 (25.0)	169 (39.49)	6118 (10.75)
65+	49,232 (87.74)	258(68.62)	112 (26.17)	49,602 (87.15)
Age at death, mean (SD)	79.58 (12.56)	72.01 (16.72)	53.60 (17.87)	79.33 (12.85)
Deaths by nationality, n (%)				
Spain	56,114 (100.0)	376 (100.0)	–	56,490 (99.25)
Africa	–	–	76 (17.76)	76 (0.13)
Asia	–	–	14 (3.27)	14 (0.02)
Eastern Europe	–	–	165 (38.55)	165 (0.29)
Latin America	–	–	84 (19.63)	84 (0.15)
Western Europe & North America	–	–	89 (20.79)	89 (0.16)
Deaths by place of birth, n (%)				
Spain	56,114 (100.0)	–	–	56,114 (98.59)
Africa	–	95 (25.27)	78 (18.22)	173 (0.3)
Asia	–	14 (3.72)	14 (3.27)	28 (0.05)
Eastern Europe	–	4 (1.06)	165 (38.55)	169 (0.3)
Latin America	–	127 (33.78)	85 (19.86)	212 (0.37)
Western Europe & North America	–	136 (36.17)	86 (20.09)	222 (0.39)

**Fig. 1:**
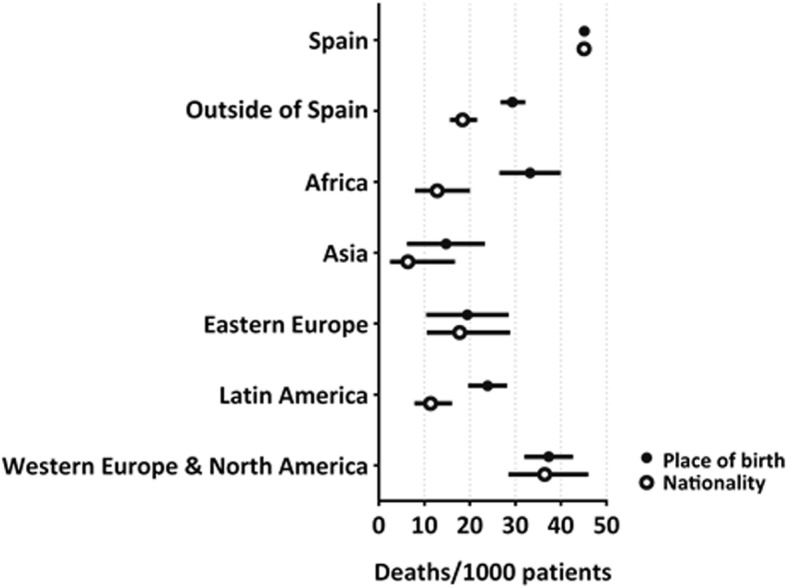
Age- and sex-standardized number of deaths per 1000 subjects between years 2012–2015 according to place of birth and nationality

Figure 1 shows the proportion of deaths between years 2012–2015 according to place of birth and nationality, standardized for age and sex. Differences were non-existent or minimal for individuals of European, North American and Asian origin, but significant for other geographical areas, with higher mortality estimates observed when immigrant status was based on place of birth versus nationality.

**Fig. 2:**
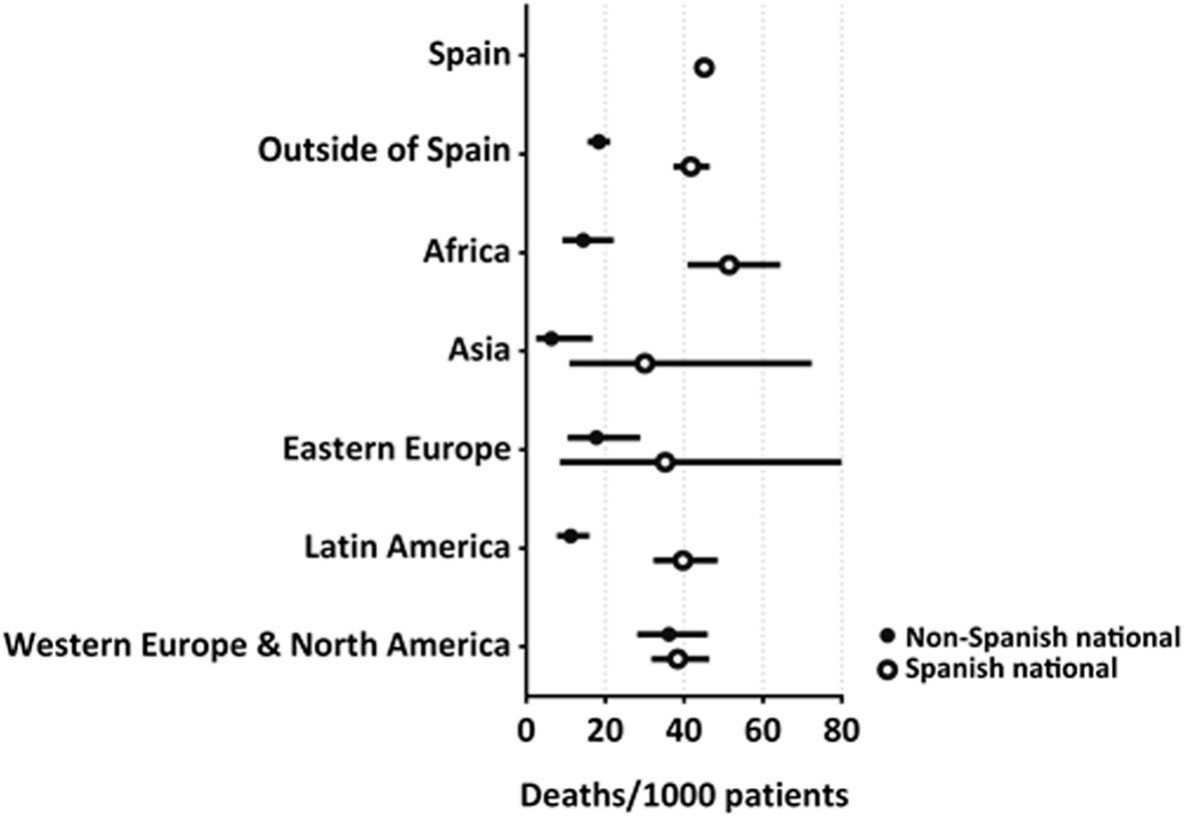
Age- and sex-standardized number of deaths per 1000 subjects between years 2012–2015 by area of birth and according to nationality

Figure 2 shows the proportion of deaths between years 2012–2015 by place of birth and according to nationality. Spanish nationals born abroad had a percentage similar to that of those born in Spain, but much higher than that of non-Spanish nationals born abroad, especially among those born in Africa and Latin America.

**Fig. 3:**
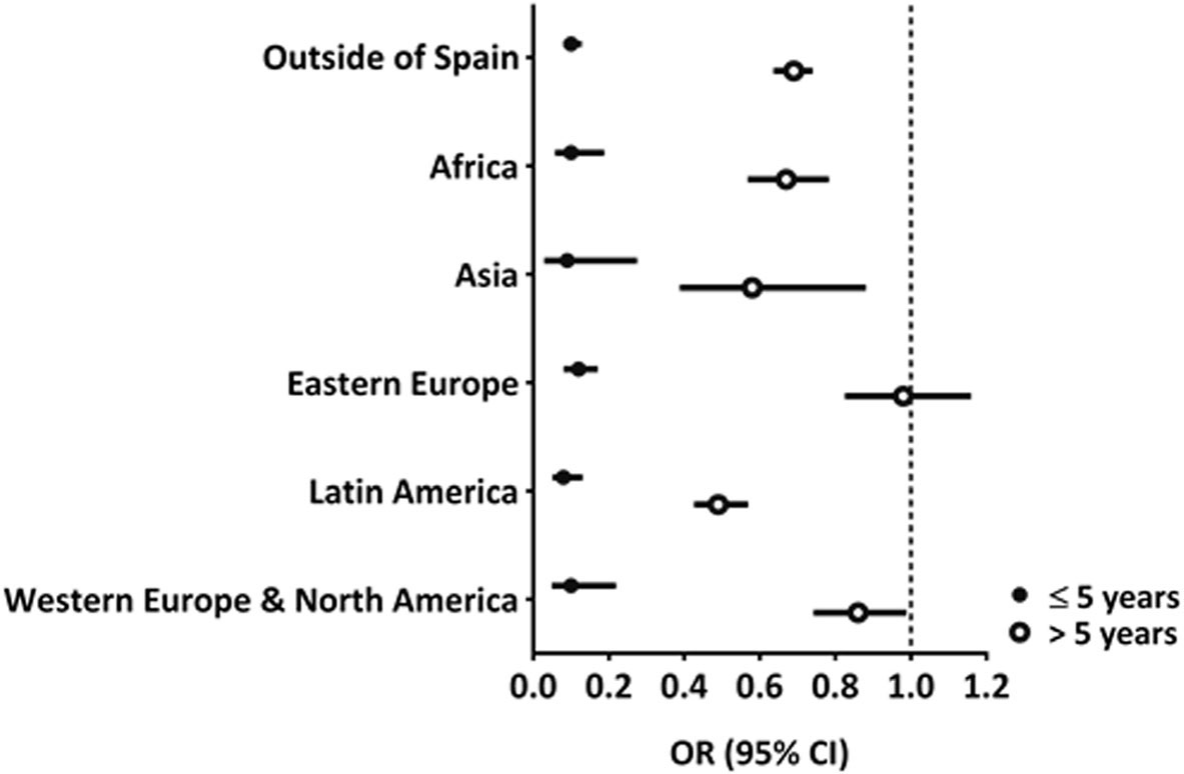
Four-year probability of death in the foreign-born versus Spanish-born population according to length of stay, adjusted by age and sex

Figure 3 shows the four-year probability of death in the foreign-born versus Spanish-born population (the comparison group is depicted using the dotted vertical line) stratified by length of stay. For the foreign-born population, regardless of the place of birth, the probability of dying during the first five years of stay in Spain was lower than that of the Spanish-born population, but this probability increased significantly when the stay exceeded five years, reaching levels comparable to those of the Spanish-born population.

**Fig. 4:**
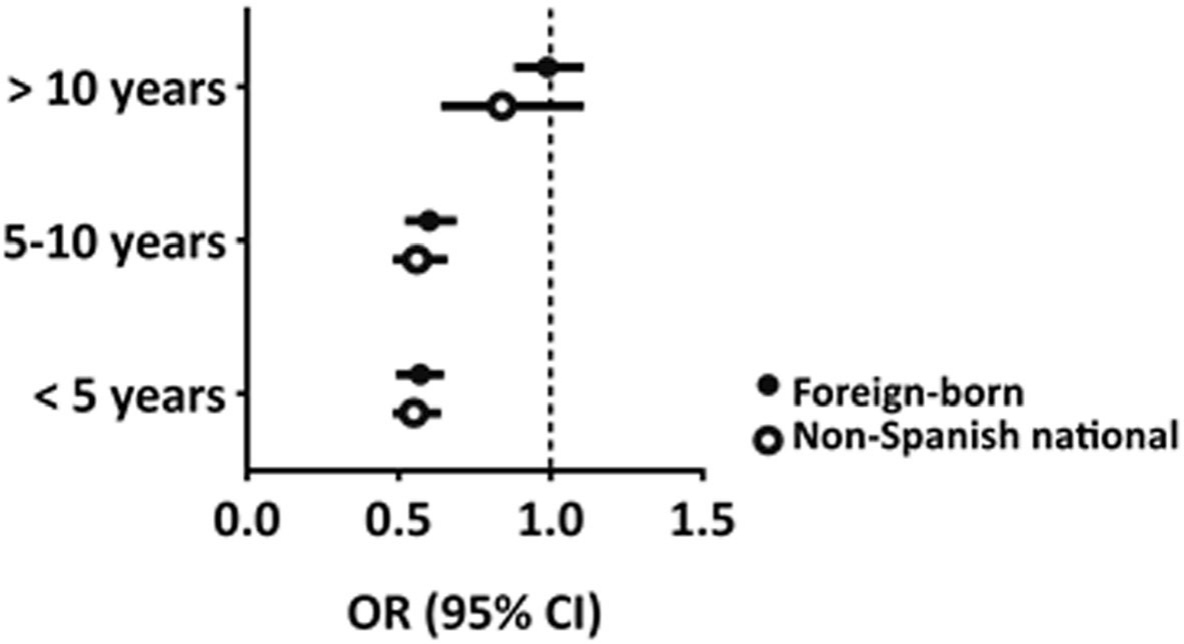
Four-year probability of death in individuals born outside of Spain and in non-Spanish nationals with respect to individuals born in Spain, stratified according to length of stay and adjusted by age and sex

Last, Fig. 4 shows the four-year probability of death according to the definition of immigrant status (place of birth versus nationality) with respect to individuals born in Spain, stratified by length of stay. Length of stay appears to be the main determinant of the risk of mortality, since the probability of dying increases with length of stay to eventually reach levels comparable to those of the Spanish-born population (i.e., > 10 years).

## Discussion

Our study reveals three important findings. First, defining immigrant status based on nationality versus place of birth results in different estimations of mortality. Second, mortality estimates of the immigrant population in Spain are lower than those of the native population, particularly on arrival, regardless of the definition used. Last, mortality increases with the length of stay, reaching a level comparable to that of the native population after 10 years.

### Practical implications of the definition of immigrant status

Our findings show that the definition of immigrant status (nationality versus place of birth) can lead to significant differences in mortality estimates. While place of birth is the criterion most frequently used [15, 19–24], immigrant status has been defined based on nationality in studies conducted in Germany [25, 26], France [27], Italy [28], Denmark [29], and Greece [30]. Several studies performed in the USA [31–34] have used the concept of ethnicity, which to date is less common in European studies. Finally, some researchers also take into account the immigration status of parents [16, 18, 35–37].

As our results indicate, the group for which the greatest disparities are observed consists of individuals whose nationality corresponds to their host country but who were born elsewhere. While multiple factors can contribute to this discordance, the following two scenarios are the most important:

1. Children born abroad with Spanish parents living abroad or mixed-nationality parents with at least one of them born in Spain. These individuals are usually not considered immigrants despite being born outside Spain, and would constitute “false positives” if considered as such.
2. Immigrants who acquire the Spanish nationality by naturalization. Failing to consider these individuals as immigrants could result in the introduction of “false negatives” into research studies. Naturalization policies are very diverse and influenced by historical, geographical, legal, and/or social factors. For example, while a non-national must, in general, reside in Spain for at least 10 years before applying for citizenship, those of Ibero-American, Andorran, Philippine, Equatorial Guinean, Portuguese, or Sephardic origin can apply after only two years.

As shown in Fig. 4, the lower death proportion observed for non-Spanish nationals born outside of Spain compared with Spanish nationals born outside of Spain appears to be due, to a large extent, to the longer duration of stay in the host country in the latter group. We therefore consider it essential that research studies take into account information relating to length of stay, in addition to nationality and place of birth, in order to avoid misinterpretation of results [12].

### Comparison of mortality estimates in native versus immigrant populations

Our results showing lower mortality in the immigrant population are in line with those of previous studies conducted in Europe [3], Norway [16, 35], Belgium [18], Spain [22], Italy [28], England [23], Canada [15, 19, 20], USA [31, 33, 34], France [24], and Denmark [29], although some studies, including one conducted in Greece [30], have reported higher mortality rates in immigrant populations.

In general, the existence of a “healthy migration effect” is accepted as a justification for the better health outcomes seen in immigrant populations. However, some morbidity studies have highlighted the possibility of a registration bias during the first years of stay of immigrants. It is proposed that, even when ill, immigrants may not avail of healthcare services due to ignorance, work priorities, language problems, or other accessibility problems [1, 7, 38]. This hypothesis is not supported by our study, since the mortality registry in Spain is exhaustive and includes data even of individuals who do not avail of healthcare services. We cannot rule out the possible existence of “salmon bias”, whereby sick migrants return to their countries of origin awaiting the outcome of disease. However, recent studies on mortality in immigrant populations have not supported this theory [23, 39].

Our results also show that mortality estimates in immigrants from Western Europe and North America are similar to those of the native Spanish population. While most immigrants from countries outside the European Union or North America come to Spain for economic reasons and end up working in unskilled jobs, those who emigrate from countries with a level of income equal to or greater than that of Spain probably work in more skilled jobs, in which health status is less important [40]. However, in the absence of socioeconomic data we cannot confirm this hypothesis.

### Influence of length of stay

The influence of length of stay in the host country on mortality has been less studied. Several studies conducted in Norway [16], Belgium [18], and Canada [15] have shown an increase in mortality with increasing length of stay. This finding has been related to the often poor socioeconomic conditions in which immigrants live [6, 17, 34], the process of acculturation [16, 18, 34], and allostatic overload [5, 38]. Our results suggest a clear temporal relationship, with length of stay in Spain constituting a “risk factor” for the health of immigrants. To some extent, immigrants “invest” their good health in improving the health of their families via the remittances they send to their countries of origin.

### Strengths and weaknesses

The strengths of this study include region-wide coverage, the inclusion of all registered immigrants and the absence of selection bias, as well as the grouping of immigrants according to area of origin, which to a certain degree recognizes the heterogeneity of the immigrant population. The use of administrative data allowed us to study the effects of important sociodemographic characteristics such as region of origin and length of stay in the host country. The selection of the outcome variable “mortality” minimizes bias associated with registration problems, since the data corresponding to this variable are obtained from a database with broad coverage and good reliability.

Some limitations to the approach used should be noted. Our study did not consider socioeconomic variables such as income, education level, legal status, or reason for migration, which could have helped identify some of the complex factors that influence mortality. This important personal information is not stored in Spanish healthcare databases and could not be obtained in any other way while preserving anonymity. Another weakness relates to our inability to take into account the “salmon bias” or “unhealthy remigration effect”.

## Conclusions

Defining immigrant status based on nationality instead of place of birth tends to result in different mortality estimates. Moreover, length of stay in the host country appears to be a key determinant of the health status of immigrants. This information should therefore be used to complement the aforementioned criteria when discriminating between the native population and non-nationals and/or foreign-born individuals. Overall, mortality estimates of immigrants are lower than those of the native population, although numbers tend to converge with increased length of stay of immigrants in the host country.

## Abbreviations

IOM: International Organization for Migration
n: Number
SD: Standard Deviation
USA: United States of America
